# Prediction of Neurocognitive Outcome after Moderate-Severe Traumatic Brain Injury Using Serum Neuron-Specific Enolase and S100 biomarkers

**DOI:** 10.25122/jml-2020-0147

**Published:** 2020

**Authors:** Dana Slavoaca, Codruta Birle, Adina Stan, Alexandru Tatomir, Oana Popa, Paula Rosu, Ana-Maria Vulcan, Diana Chira, Livia Livint Popa, Constantin Dina, Vitalie Vacaras, Stefan Strilciuc, Pieter Vos

**Affiliations:** 1. Department of Neurosciences, “Iuliu Hatieganu” University of Medicine and Pharmacy, Cluj-Napoca, Romania; 2. “RoNeuro” Institute for Neurological Research and Diagnostic, Cluj-Napoca, Romania; 3. Department of Neurology, University of Maryland, School of Medicine, Baltimore, United States of America; 4. Department of Radiology, “Ovidius” University, Faculty of Medicine, Constanta, Romania; 5. Department of Neurology, Slingeland Hospital, Doetinchem, The Netherlands; 6. Neurology Clinic, Cluj Emergency County Hospital, Cluj-Napoca, Romania

**Keywords:** Traumatic Brain Injury, NSE, S100, Neurocognitive Outcome, ECLIA - electrochemiluminescence immunoassay analyzer, ERBI - Early Rehabilitation Barthel Index, GCS - Glasgow Coma Scale, GOSE - Glasgow Outcome Scale Extended, HADS - Hospital Anxiety and Depression Scale, MMSE - Mini-Mental State Examination, NSE - Neuron-Specific Enolase, PSI - Processing Speed Index, RMSEA - Root Mean Square Error of Approximation, SEM - Structural Equation Modeling, TBI - Traumatic Brain Injury, WAIS - Wechsler Adult Intelligence Scale

## Abstract

Seric biomarkers have been tested in a large number of studies on traumatic brain injuries (TBI) patients in order to predict severity, especially related to the short-term outcome. However, TBI patients have a high risk of developing long-term complications such as physical disability, cognitive impairment, psychiatric pathology, epilepsy, and others.

The aim of this study was to assess the correlation between protein biomarkers S100 and neuron-specific enolase (NSE) and neurocognitive status at 10- and 90-days post-injury.

Both biomarkers were tested in the first 4h and after 72h post-injury in 62 patients with moderate-severe TBI. The patients were evaluated by a series of neurocognitive tests: Early Rehabilitation Barthel Index (ERBI), Glasgow Outcome Scale-Extended (GOSE), The Mini-Mental State Examination (MMSE), Processing Speed Index (PSI), and Stroop Test, at 10 and 90 days post-injury and supplementary by the Hospital Anxiety and Depression Scale at 90 days. For evaluating the whole neurocognitive status instead of every scale separately, we used Structural Equation Modeling (SEM), while for anxiety and depressive symptoms, we used multiple regression analyses. SEM showed that NSE values at 4 hours were significant predictors of the cognitive status at 10 (p=0.034) and 90 days (p= 0.023). Also, there were found significant correlations between NSE at 4h and the anxiety level. This study demonstrated a significant correlation between NSE at 4h and short and medium-term neuropsychological outcomes, which recommends using this biomarker for selecting patients with a higher risk of cognitive dysfunction.

## Introduction

The outcome in traumatic brain injuries (TBI) is essential both for identifying patients with a higher risk of unfavorable short-term outcomes – death, physical disability, and selecting patients at risk for developing long-term complications. Patients with severe TBI are prone to different levels of neurocognitive dysfunction, the most affected being working memory, attention, information processing, cognitive flexibility and learning capability [1]. Even patients with mild TBI may temporarily develop neurocognitive deficits in the first months after the injury, but the deficits are generally remitted in the first six months [2]. History of TBI has been associated with an increased risk of cognitive decline in older adults and an earlier age of dementia onset [3, 4]. The risk factors for long-term cognitive impairment in patients with TBI include the severity of the injury [5], old age [6], length of hospital stay [7] and preinjury educational level [8]. Selecting the patients at risk of developing cognitive impairment after TBI is crucial for establishing a suitable treatment strategy, a personalized rehabilitation program, and communicating with the patients and their relatives. However, prediction of outcome in TBI represents a real challenge due to the complexity of factors that interact at different levels in the specific physiopathological processes and the phenotypic heterogeneity of the injury.

Serial blood-derived biomarkers have been intensively studied, especially as assessment and screening tools. Biomarkers are useful for selecting patients with higher chances for having intracerebral lesions, and as prognostic tools for assessing mortality and severe disability, usually related to the short-term outcome [9]. The most used methodology consists of testing one or several biomarkers, measured once, usually in the acute phase, and classifying outcome as either favorable or unfavorable. However, recent studies propose a more complex approach, either by monitoring the biomarkers’ level at several points in time and highlighting their dynamics in time or using composite biomarkers [9–11]. Some studies also tested the correlation between biomarker levels and cognitive function, usually assessed at 3 or 6 months. Several biomarkers were tested, the most used being S100B, and correlated separately with different scales representing different cognitive domains, with promising results [12–14]. A recently published paper has found a significant correlation between higher levels of a broad panel of pro-inflammatory interleukins and lower scores in Glasgow Outcome Scale-Extended (GOSE) at three and six months and the California Verbal Learning Test at six months [11].

This study aimed to test an affordable approach to selecting patients more prone to long-term cognitive impairment. Two biomarkers that are easy to use in clinical practice - S100 and Neuron-Specific Enolase (NSE), were tested, both at 4h and 72h post-injury. Serum levels of NSE and S100B at 4h and 72h post-injury were correlated with the neurocognitive status, captured by an ensemble of outcome scales at 10 and 90 days. By using a multidimensional approach to evaluate the outcome at two different points in time, this study tried to capture a potential correlation between these biomarkers and the global status of TBI patients.

## Material and Methods

### Study population

A subgroup of 62 patients was selected from a total group of 132 patients with moderate-severe TBI (GCS 7-12) included in the CAPTAIN II trial [15]. The study was approved by the Ethics Committee of the University of Medicine and Pharmacy in Cluj-Napoca, Romania (No. 714/07.03.2013). Because of different outcome evolution between the treatment group and placebo, the patients selected for these analyses were those without active study medication.

### Sample collection

Biomarkers’ sampling was performed at 4h and 72h after the injury. The S100 and NSE levels were measured using an electrochemiluminescence immunoassay analyzer (ECLIA) from a sample of 0.5 mL of venous blood centrifugated at 1500 rpm for 10 minutes at 4°C. After centrifugation, the supernatant was kept frozen at -80°C until analyses. The laboratory upper limits are 0.105 μg/L3 for S100 and 16.3 ng/mL for NSE.

### Outcome measures

The evaluations performed at 10 and 90 days were selected from the CAPTAIN II dataset [15]. At both visits, the following scales were assessed:

1. Glasgow Outcome Scale-Extended (GOS-E) [16], 10- and 90-days visits;
2. Early Rehabilitation Barthel Index (ERBI) [17], 10- and 90-days visits;
3. Mini-Mental State Examination (MMSE) [18], 10- and 90-days visits;
4. Processing Speed Index (PSI), Wechsler Adult Intelligence Scale (WAIS) [19], two subscales, 10- and 90-days visits;
5. Stroop Color-Word Test—Victoria Version (VST) [20], three subscales, 10- and 90-days visits;
6. Hospital Anxiety and Depression Scale (HADS) [21], two subscales, 90 days visit.

### Statistical analyses

We analyzed the correlation between S100B and NSE in the first 4h and 72h post-TBI and the ensembles of neuro-psychological scales at 10 and 90 days. Before analysis, data were examined for accuracy of data entry, missing values, normality, and outliers.

A preliminary analysis of paired sample t-test was conducted to investigate changes in scores between the two-time points. Effect sizes were indicated by Cohen’s d (small effect = 0.2; medium effect = 0.5, large effect = 0.8).

**Figure 1:**
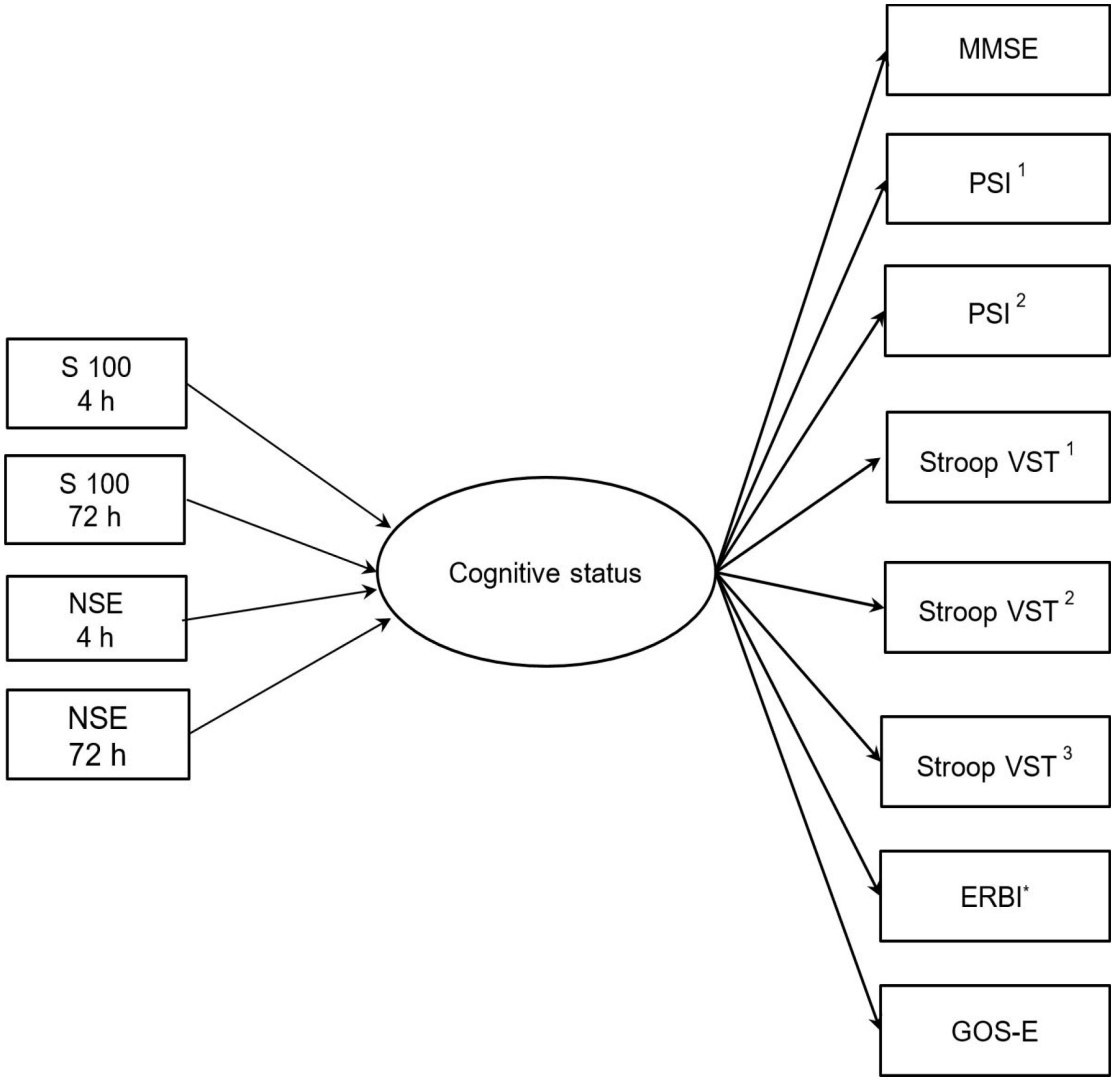
Illustration of measurement and structural models tested twice at 10 and 90 days.

Taking into account the physiological overlap between cognitive subdomains [22] and the concept analyzing the relations among serum biomarker levels and cognitive status, a structural equation modeling (SEM) approach was utilized. Subsequently, a two-stage analytical approach [22] was used, including the measurement model first, followed by the structural model (Figure 1).

Confirmatory factor analysis was conducted using the Maximum Likelihood estimator. The measurement model included one latent variable, neurocognitive status, defined from the observed scores on the following scales: (1) MMSE; (2) PSI – 2 subscales; (3) Stroop Color-Word Test – 3 subscales; (4) GOS-E; (5) ERBI. The structural model included observed values of the serum biomarkers S100 and NSE at 4h and 72h as exogenous variables and latent cognitive status as an endogenous variable. Measurement and structural models were run separately to reflect measures at 10 and 90 days. Overall model fit was assessed using the following indices [23]: χ2/df ratio lower than 3, Comparative Fit Index (CFI ≥ 0.90), Root Mean Square Error of Approximation (RMSEA < 0.08), and Standardized Root Mean Square Residual (SRMR < 0.08). SEM analyses were run using the lavaan package for R [24].

Relations among serum levels and emotional symptoms were investigated using regression analysis. Two multiple linear regression analyses were run to predict anxiety and depression scores, as reflected by the Hospital Anxiety and Depression Scale at 90 days and based on S100 and NSE values at 4h and 72h, respectively (uncontrolled for other variables). Standardized beta coefficients are reported (variances of dependent and independent variables 1).

## Results

In the first 4h after injury, median serum concentrations were: S100=0.52 μg/l (sd=0.30) and NSE=22.54 ng/dl (sd=18.46). At 72h post TBI, S100 and NSE decreased to 0.20μg/l (sd=0.30) and 15.3ng/l (sd=8.60), respectively.

### Preliminary results

Means and standard deviations of the cognitive measures are shown in Table 1. Paired sample t-tests indicated significant differences between all measures at 10 and 90 days. The direction of change was indicative of cognitive improvement for all measures. There was an increase in scores for MMSE (d = 1.21); PSI1 (d = 1.31), PSI2 (d = 1.41), GOS-E (d = 1.39) and ERBI (d = 0.30), as well as a decrease in scores for all Stroop measures, VST 1(d = 1.45), VST 2 (d = 1.86 ), VST 3 (d = 1.55).

**Table 1:** Descriptive statistics and paired t-tests.

	10 days Mean (SD)	90 days Mean (SD)	t-test (p value)
**MMSE**	25.92 (3.94)	29.01 (1.57)	t(53) = -8.28 p < .001
**PSI^1^**	42.46 (13.05)	49.33 (13.10)	t(53) = -9.82 p < .001
**PSI^2^**	19.61 (6.79)	27.62 (6.78)	t(53) = -10.47 p < .001
Stroop VST^1^	23.31 (5.68)	18.64 (6.28)	t(53) = 10.76 p < .001
**Stroop VST^2^**	33.90 (8.32)	27.61 (8.52)	t(53) = 14.04 p < .001
**Stroop VST^3^**	59.50 (20.12)	51.88 (19.75)	t(53) = 13.27 p < .001
**GOS-E**	6.16 (1.24)	7.40 (0.78)	t(53) = -2.26 p < .05
**ERBI**	92.59 (24.02)	100 (0.00)	t(53) = -10.25 p < .001

Note: SD = standard deviation; PSI1 = PSI Digit Symbol Coding; PSI2 = PSI Symbol Search; Stroop VST 1 = Stroop Version Dots; Stroop VST 2 = Stroop Version Words; Stroop VST 3 = Stroop Version Colors.

### Serum biomarker levels and cognitive status

The results indicated that the measurement models fitted the data well, for cognitive status at 10 days (χ2/df = 1.38, CFI = 0.987, RMSEA = 0.084 [0.000, 0.167], SRMR = 0.038), and 90 days (χ2/df = 0.72, CFI = 1.00, RMSEA = 0.000 [0.000, 0.104], SRMR = 0.022) (Table 3). ERBI scores at 90 days presented zero variance and were therefore not included in the 90 days measurement model. Both models were retained in subsequent modeling.

**Table 2:** Measurement model at 10 and 90 days. It can be observed that even though there is a good generally correlation between scales, the most decreased factor loading is related to GOSE.

Latent variables	Estimate	Std. Err	P(>|Z|)	Std. all
**Cognitive ability 10 days**				
MMSE	1.000			0.901
PSI^1^	3.192	0.348	0.000	0.883
PSI^2^	1.567	0.179	0.000	0.864
STROOP^1^	-1.144	0.164	0.000	-0.772
STROOP^2^	-1.931	0.220	0.000	-0.866
STROOP^3^	-4.457	0.513	0.000	-0.887
ERBI	3.988	0.754	0.000	0.592
GOSE	0.277	0.034	0.000	0.838
Cognitive ability 90 days				
MMSE	1.000			0.817
PSI^1^	7.676	1.252	0.000	0.771
PSI^2^	3.662	0.645	0.000	0.729
STROOP^1^	-3.980	0.558	0.000	-0.856
STROOP^2^	-5.380	0.775	0.000	-0.841
STROOP^3^	-13.851	1.698	0.000	-0.938
GOSE	0.431	0.076	0.00	0.729

**Table 3:** Standardized regression paths (SEM).

	Cognitive status 10 days		Cognitive status 90 days	
	b	p-value	b	p-value
S100 4h	0.072	0.660	0.136	0.396
S100 72h	-0.095	0.515	-0.213	0.138
NSE 4h	-0.388	0.034	-0.411	0.023
NSE 72h	-0.052	0.792	-0.067	0.729

Note: β = standardized regression coefficient; significant effects indicated in bold.

The results also showed that the specified structural model fitted the data well at 10 days (χ2/df = 1.42, CFI = 0.956, RMSEA = 0.094 [0.026, 0.145], SRMR = 0.063) and 90 days (χ2/df = 1.38, CFI = 0.955, RMSEA = 0.090 [0.000, 0.147], SRMR = 0.071).

As shown in Table 2, NSE values at 4 hours were significant predictors of the cognitive status at 10 and 90 days. As reflected in these results, higher NSE values at 4h were indicative of worse short- and medium-term cognitive functioning. The remaining paths were not significant.

### Serum biomarkers and emotional symptoms

**Figure 2:**
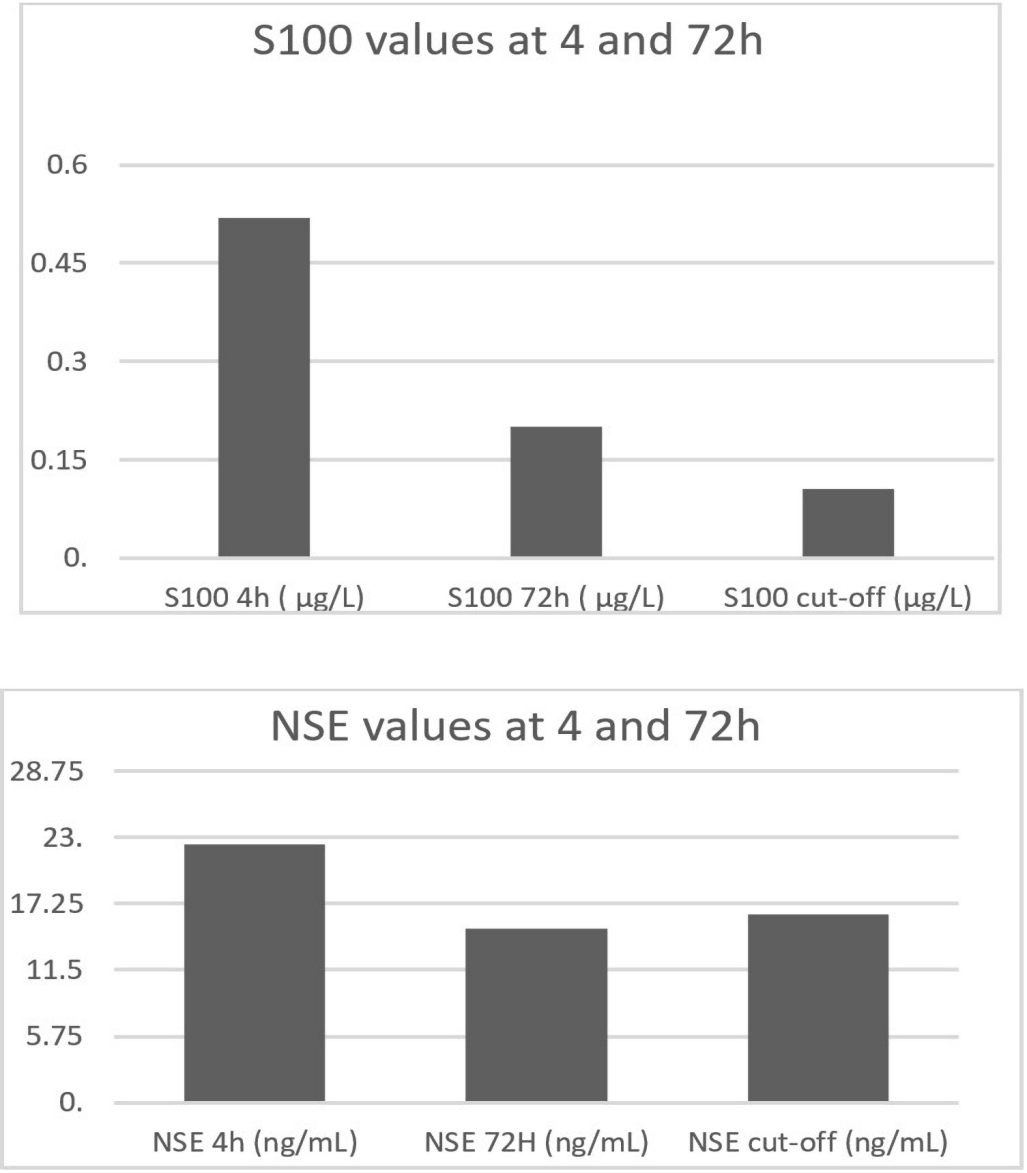
Comparison between S100 and NSE values at 4h and 72h, versus laboratory cut-off.

A multiple regression analysis was run to predict anxiety scores based on S100 and NSE values, at 4h and 72h, respectively (Figure 2). The results indicated a collective significant effect, F (4, 43) = 2.81, p=0.037, r2=.20. The examination of individual predictors showed that NSE at 4 hours was the only significant predictor in the model (β= 0.40, t=2.196, p=0.034). As reflected in these results, higher NSE at 4h was indicative of higher medium-term anxiety.

A multiple regression analysis was run to predict depression scores based on S100 and NSE values, at 4h and 72h, respectively. The results indicated that none of the predictors had a significant effect on the outcome measure, F(4, 43) = 2.16, p=0.089.

## Discussion

The purpose of this study was to examine the ability of serum S100 and NSE concentration in the first 4h and at 72h to predict the neuropsychological outcome at 10 and 90 days post-TBI.

A strength of this study is that the neuro-cognitive status was analyzed as a whole, instead of establishing a correlation between biomarkers with each scale separately. The rationale behind this decision was driven by the need for capturing the multidimensionality of the patient’s full status [25]. Moreover, there is a well-established overlap and interaction between cognitive processes, supported by the dynamics of neuronal networks [26, 27]. In this study, we analyzed, using the SEM approach, the correlation between biomarkers and different cognitive components. We focused mainly on executive functions, such as working memory, speed processing, perceptual processing [28], response inhibition and selective attention [29], together with assessment scales of general outcome such as GOSE and ERBI. SEM represents a mathematical modeling approach that has two parts: a measurement model and a structural model. The measurement model describes the relationship between the observed variables (in this case, S100 and NSE) and the latent factors (the results from the neurocognitive tests). The structural model is a regression model in which latent variables can be predicted by other manifest or latent variables [30–32]. We found a good factor loading between all neurocognitive scales, except for GOSE. Even though it has an increased complexity than the initial GOS, GOSE still divides the outcome into broad categories and has a low capacity in discriminating between physical and cognitive-emotional disabilities [33]. Therefore, the decreased GOSE’s factor loading can be explained by its reduced capacity of capturing the complexity of TBI outcome.

Neurocognitive outcome at 90 days was improved compared to the results at 10 days, similar to the results from other studies [34–36]. Both biomarkers showed a descending trend from the first 4h post-TBI to 72h, which is in accordance with the literature data regarding the timeline evolution of these two biomarkers [37–39]. According to data from other studies, S100B has two peaks, first in the first 6h and the second one at 24h, the second one having a stronger predictive power than the first one. The blood half-life appears to be at 24h, but some studies showed more unpredictable dynamics, with several peaks and rapid decreases [40, 41]. There is no known information explicitly related to S100. Our data showed higher serum levels than the laboratory cut-off at both 4h and 72h, even there was a significant decrease between these two-time points. There are fewer data about NSE, in comparison with S100B. However, other studies reported an initial increase in the first hours and normalization of serum levels after 24-48h post-TBI [40], which is in accordance with our results.

S100 represents a calcium-binding protein family with various roles in cellular processes. In relation to TBI, the most studied parameter is S100B, which is considered a parameter of astrocytes’ damage [42]. In contrast with S100B, there are sparse data on S100 in TBI studies. To our knowledge, there are three studies in relation to cognitive function, all of them showing positive correlations between lower S100 serum levels and better cognitive results. Contrary to these previous results [13, 43, 44], we have not found any significant correlation between this biomarker and neuro-psychological status at neither 4h nor 72h post-injury. A potential explanation could be the different periods post-injury of biomarker testing and the already recorded unpredictability of the dynamics of this biomarker. Serum levels at 4h may also reflect other sources of S100, especially since S100 has lower specificity than S100B.

The only correlation that we found was between NSE at 4h and the neuro-cognitive status at both 10 and 90 days. Even that NSE had been previously correlated with unfavorable outcomes [45], to our knowledge, this is the second study that analyses the correlations of NSE levels and neuropsychological status of TBI patients [44] and the first one that included only moderate-severe patients. NSE was correlated not only with the neurocognitive outcome but also with anxiety scores. The correlation is in accordance with other data in the literature that showed overlap between anxiety and cognitive function [46, 47]. The positive correlations of NSE with both short-term and medium-term neuro-cognitive outcome suggests that NSE can be used for selecting patients more prone to cognitive dysfunction, results supported by the data related to higher NSE values in other pathologies associated with the development of cognitive impairment [48, 49].

## Conclusions

In this study, we analyzed serum biomarkers S100 and NSE, starting with the first 4h post-TBI. The lack of correlation between S100 and neurocognitive outcomes suggests that this biomarker probably has a better prediction value between 12 and 24h. The dynamic pattern of NSE, along with a good correlation at 4h post-injury with the neurocognitive outcome combined with lower serum values at 72h than the laboratory cut-off, suggests a narrow timeline of this biomarker.

In this study, we used a dynamic evaluation in time (at 10 and 90 days) supported by a complex battery of tests to capture as much information as possible from the neuropsychological status of TBI patients. For analyzing the neurocognitive status as a unitary outcome, we used MSE, which showed a generally good correlation between neurocognitive tests, with a lower factor loading for GOSE. We found a significant correlation between NSE at 4h and short and medium-term neuropsychological outcomes, which recommends using this biomarker for selecting patients with a higher risk for cognitive dysfunction.

## Conflict of Interest

The authors declare that there is no conflict of interest.
